# Treatment of vascular dementia. Recommendations of the Scientific Department of Cognitive Neurology and Aging of the Brazilian Academy of Neurology

**DOI:** 10.1590/S1980-57642011DN05040005

**Published:** 2011

**Authors:** Sonia Maria Dozzi Brucki, Ana Cláudia Ferraz, Gabriel R. de Freitas, Ayrton Roberto Massaro, Márcia Radanovic, Rodrigo Rizek Schultz, Working Group on Alzheimer's Disease and Vascular Dementia of the Brazilian Academy of Neurology

**Affiliations:** 1 Neurology Service, Hospital Santa Marcelina, Cognitive and Behavioral Neurology Group of Clínicas Hospital of the University of São Paulo School of Medicine (FMUSP), Referral Center for Cognitive Disorders (CEREDIC) of the FMUSP, São Paulo SP, Brazil; 2 D'Or Institute of Research and Teaching, University Federal Fluminense, Rio de Janeiro RJ, Brazil; 3 Institute of Rehabilitation Lucy Montoro; 4 Medical Investigation Laboratory 27 (LIM 27), Institute of Psychiatry, School of Medicine, University of Sao Paulo, São Paulo SP, Brazil; 5 Sector of Behavioral Neurology of the Department of Neurology and Neurosurgery of the Federal University of São Paulo (UNIFESP), Center for Brain Aging (NUDEC) - Institute of Memory (UNIFESP), São Paulo SP, Brazil

**Keywords:** vascular dementia, pharmacological treatment, prevention, cholinesterase

## Abstract

Scientific Department of Cognitive Neurology and Aging of ABN had a consensus meeting to write recommendations on treatment of vascular dementia, there was no previous issue. This disease has numerous particularities and can be considered a preventable dementia. Prevention treatment is primary care of vascular risk factors or a secondary prevention of factors that could cause recurrence of ischemic or hemorrhagic brain modifications. In these guidelines we suggested only symptomatic treatment, pharmacologic or non-pharmacologic. We have reviewed current publications on MEDLINE (PubMed), LILACS e Cochrane Library databases. Recommendations are concern to the following factors and their prevention evidences, association, or treatment of vascular dementia: physical activity, tobacco use, diet and food supplements, arterial hypertension, diabetes mellitus, obesity, statins, cardiac failure, atrial fibrillation, antithrombotics, sleep apnea, carotid revascularization, symptomatic pharmacological treatment.

## Abstract (pt)

O DC de Neurologia Cognitiva e do Envelhecimento da ABN reuniu-se para escrever recomendações para o tratamento da demência vascular, uma vez que não havia nenhuma recomendação neste sentido. Esta doença tem inúmeras particularidades e pode ser considerada uma das demências que pode ser prevenida. O tratamento para sua prevenção é o de cuidados primários para os fatores de risco vasculares, ou a prevenção secundária dos fatores que levam a recorrência de alterações isquêmicas ou hemorrágicas cerebrais. Nestas recomendações sugerimos apenas o tratamento sintomático, medicamentoso ou não. Baseamo-nos nas evidências disponíveis, através da revisão das publicações nas bases MEDLINE (PubMed), LILACS e Cochrane Library. As recomendações dizem respeito aos seguintes fatores e suas evidências na prevenção, associação ou tratamento da demência vascular: atividade física, álcool, tabagismo, dieta e suplementos, hipertensão arterial, diabetes mellitus, obesidade, estatinas, insuficiência cardíaca, fibrilação atrial, antiagregantes, apneia do sono, revascularização carotídea e tratamento farmacológico sintomático.

The diagnosis of vascular cognitive impairment (VCI) and vascular dementia (VD) remains controversial. Since a number of criteria are available in the literature, this discussion is of primary importance to Brazil in furthering knowledge, improving diagnosis and gleaning greater understanding of the mechanisms involved in the emergence of cognitive decline due to vascular causes in the Brazilian population.

Thus, VCI has recently been proposed as a term encompassing VD and all other forms of mild to severe cognitive impairment secondary to cerebrovascular disease. The term encompasses three conditions: VCI no dementia, vascular dementia, and AD with a vascular component. VCI no dementia constitutes the most prevalent VCI subgroup in persons younger than 85 years of age. VD denotes dementia caused by all types of vascular pathology. The current classification of VD includes cortical vascular dementia, subcortical ischemic VD, dementia due to strategic infarct, dementia due to hypoperfusion, and dementia due to hemorrhagic lesions.

Therefore, this subject was extensively addressed and a consensus on its diagnostic criteria by this group can be found in an earlier publication.

Given the array of (often imprecise) criteria used to diagnose VCI and VD among studies on the treatment of vascular risk factors, the task of defining recommendations for treating these diseases, particularly in the prevention of cognitive decline, does not have robust levels of evidence to draw on.

The heterogeneity of the physiopathology, location and magnitude of lesions, diagnostic criteria, and cognitive assessment all vary among studies on cognition, precluding, in the majority of cases, generalization of results for all types of impairment.

## Methods

A search of the electronic PubMed and Scielo databases for articles published up to May 2011 was carried out. Studies containing an abstract that addressed the association among VCI and VD, symptomatic treatment of risk factors for vascular diseases and cognition, or specific treatment of cognition-related symptoms in patients with VCI or VD, were included. Review type articles and meta-analyses on the theme were also included.

The themes chosen were divided by members of the consensus group into: physical activity; diet and food supplementation; alcohol, obesity, smoking, treatment of arterial hypertension, diabetes, dyslipidemia, cardiac insufficiency, atrial fibrillation;sleep apnea and antiaggregants. Regarding specific symptomatic treatment, trials with cholinesterase inhibitors and glutamate antagonist, cytocholine, calcium channel blockers, cerebrolysin and pentoxifylline were assessed.

After selection of articles, these were classified into Classes I, II and III, while recommendations were qualified according to levels of evidence with A, B, C and U ratings. The criteria employed are summarized in Tables 1 and 2, and were based on the recommendations of the American Academy of Neurology published in 2008.

**Table 1:** Classification of studies.

Class I.	A randomized, controlled clinical trial of the intervention of interest with masked or objective outcome assessment, in a representative population. Relevant baseline characteristics are presented and substantially equivalent among treatment groups or there is appropriate statistical adjustment for differences. The following are also required:		
	a.	Primary outcome(s) clearly defined.	
	b.	Concealed allocation clearly defined.	
	c.	Exclusion/inclusion criteria clearly defined.	
	d.	Adequate accounting for drop-outs (with at least 80% of enrolled subjects completing the study) and cross-overs with numbers sufficiently low to have minimal potential for bias.	
	e.	For non-inferiority or equivalence trials claiming to prove efficacy for one or both drugs, the following are also required*:	
		1.	The standard treatment used in the study is substantially similar to that used in previous studies establishing efficacy of the standard treatment. (e.g. for a drug, the mode of administration, dose and dosage adjustments are similar to those previously shown to be effective).
		2.	The inclusion and exclusion criteria for patient selection and the outcomes of patients on the standard treatment are comparable to those of previous studies establishing efficacy of the standard treatment.
		3.	The interpretation of the results of the study is based upon an analysis of cases observed.
**Class II.**	A randomized controlled clinical trial of the intervention of interest in a representative population with masked or objective outcome assessment that lacks one criteria a-e above or a prospective matched cohort study with masked or objective outcome assessment in a representative population that meets b-e above. Relevant baseline characteristics are presented and substantially equivalent among treatment groups or there is appropriate statistical adjustment for differences.		
**Class III.**	All other trials (including well-defined natural history controls or patients serving as own controls) in a representative population, where outcome is independently assessed, or independently derived by objective outcome measurement.		
**Class IV.**	Studies not meeting Class I, II or III criteria including consensus or expert opinion.		

* Note that numbers 1-3 in Class Ie are required for Class II in equivalence trials. If any one of the three are missing, the class is automatically downgraded to Class III.

**Table 2:** Levels of evidence.

**A.**	Established as effective, ineffective or harmful (or established as useful/predictive or not useful/predictive) for the given condition in the specified population (Level A rating requires at least two consistent Class I studies)*.
**B.**	Probably effective, ineffective or harmful (or probably useful/predictive or not useful/predictive) for the given condition in the specified population (Level B rating requires at least one Class I study or two consistent Class II studies).
**C.**	Possibly effective, ineffective or harmful (or possibly useful/predictive or not useful/predictive) for the given condition in the specified population (Level C rating requires at least one Class II study or two consistent Class III studies).
**U.**	Data inadequate or conflicting;given current knowledge, treatment (test, predictor) is unproven.

* In exceptional cases, one convincing Class I study may suffice for an "A" recommendation if 1) all criteria are met, 2) the magnitude of effect is large (relative rate improved outcome > 5 and the lower limit of the confidence interval is > 2).

### Physical activity

Animal studies have shown that physical activity stimulates angiogenesis, synaptogenesis and neurogenesis. Rats subjected to treadmill running had more astrocytes and neuroblasts able to proliferate in the subgranular zone of the dentate gyrus of the hippocampus, as well as a greater number of neurons in the transient stage, compared to control animals.^1^

Physical exercise also reduces risk factors for vascular diseases and can release hormonal factors which enhance neuronal functioning, lending support to the theory of potential benefits. Such benefits have also been confirmed in clinical trials.

In a recent 8-year follow-up study involving 3075 elderly between 70 and 79 years of age in 1997, 30% of the group presented cognitive decline at the end of the follow-up period. Multivariate analysis of the initial variables associated good evolution with younger age, white ethnicity, higher level of education and literacy, moderate to vigorous exercise, and non-smoking status.^2^

Some evidence indicates that physical activity plays a role in preventing dementia and conversion from mild cognitive impairment (MCI) to dementia in the form of Alzheimer's disease (AD) or VD.^3-6^ A recent meta-analysis of prospective studies involving 33816 non-demented individuals at base line, followed up for one to 12 years, found that a high level of physical activity was associated to a 38% reduction in risk of cognitive decline, whereas light to moderate physical activity was also associated to a 35% reduction in risk of decline.^7^

In the randomized clinical trial by Lautenschlager et al., physical activity was associated to lower risk of developing MCI and dementia among adults with subjective memory complaints.^3^ Volunteers were 50 years of age or older and randomly assigned to an educational program or to a domiciliary physical exercise program lasting a period of 24 weeks. A statistically significant difference, albeit modest, was seen in the cognitive performance of the group that performed the physical activity, a difference which persisted after an 18-month period.^3^

Studies in the literature show a statistically significant benefit from physical activity. However, preventive effects seem to be weaker in VD than AD.^8^ Studies investigating cognitive performance and physical activity have tended to involve a smaller number of VD patients, yet all showed a reduced risk of dementia.^9,10^

In post-stroke patients, there is an increased risk for reduced physical activity, predominantly among those with cognitive impairment, especially executive function disorders.^11^

A meta-analysis revealed lower VD in patients that performed physical activity (odds ratio 0.62 - CI: 0.42-0.92).^12^ In an observational study in community-dwelling elderly, physical activity proved preventive for the development of VCI in women.^13^ An Italian prospective study assessing the efficacy of physical activity in reducing the risk of developing AD or VD in elderly individuals found physical activity to be associated to a lower risk of VD, but not of AD.^14^ A prospective long-term (up to 21 years) follow-up study of 401 community-dwelling older adults, found that taking part in cognitive activities, but not in physical activities, was associated to a lower risk of VCI with or without dementia.^15^

Several controlled trials are underway assessing the effect of physical activity on patients with VCI and AD.^16,17^

**Recommendations** - Regular physical activity should be recommended to healthy individuals, patients with cerebrovascular disease, and to patients with cognitive decline (Level of evidence B)

### Diet and supplements

A balanced diet, specifically a Mediterranean diet characterized by high consumption of fruit, vegetables, legumes, grains and unsaturated fatty acids (olive oil);low intake of milk and milk-derived products, meat and saturated fatty acids plus moderate consumption of alcohol, have been associated to a lower risk for dementia as well as reduced conversion of MCI to AD.^18-20^ The vascular factors can be linked to a Mediterranean diet, but other non-vascular biological mechanisms (oxidative and inflammatory) may be responsible for explaining the complex epidemiological association between a Mediterranean diet and cognitive decline.^21^

In a population-based cohort study, high adherence to a Mediterranean diet was associated, during follow up, with lower decline in scores on the Mini-Mental State Exam, although this did not translate to a similarly reduced risk for dementia. Differences can occur depending on the study venue and on previous diet, whereby the cited study was run in France, whilst the others were done in North America.^22^

Randomized trials have failed to show any effect of food supplementation with various substances on cognitive decline prevention. Substances supplemented have included omega 3;^23,24^ vitamin C, E and beta carotene,^25^ vitamin B12, folic acid and vitamin B6.^26^

**Recommendations** - Adapting the diet and effecting changes in eating habits are important by promoting consumption of healthy foods predominantly vegetables, unsaturated fatty acids, grains and fish (Level of evidence B).

### Alcohol

The majority of studies on alcohol and cognition have shown that consumption of low amounts of alcohol has a preventive effect on the development of VD, AD and other types of dementia.^27^

There is compelling evidence that consumption of alcohol in moderation is associated to a lower risk of coronary diseases, ischemic strokes and dementia. The underlying protective mechanisms involved include reduced LDL and increased HDL;decreased resistance to insulin; lowering of blood pressure; reduced platelet aggregation and fibrinogen levels and lower serum homocysteine and inflammatory markers. In addition, anti-amyloidogenic activity promoted by resveratrol (present in red wine) also seems to occur.^28-35^

Although studies show a positive correlation between alcohol consumption and dementia prevention, with some benefit associated to wine consumption, there is a deleterious effect of alcohol when consumed at high doses. The consumption of two daily drinks (<30 g/d) is associated with reduced overall risk, whereas three or more drinks is associated to increased risk of ischemic or hemorrhagic stroke (CVA).^36^

**Recommendations** - The consumption of high doses of alcohol must be avoided (Level of evidence C).

### Obesity

Dementia risk is higher among overweight and obese individuals.^2-37^ There is a positive association between body mass index in adult life and emergence of AD and VD in later life. with a five-fold higher risk of VD in obese subjects and twice the risk among overweight individuals, independently of vascular factors.^38^

**Recommendations** - Keeping weight within normal levels should be encouraged (Level of evidence C).

### Systemic arterial hypertension

Systemic arterial hypertension (SAH) is a potential risk factor for cognitive impairment and dementia, including the vascular type.^39^ A class-specific effect of anti-hypertensive drugs toward reducing cognitive impairment has been postulated in some studies. However, the cognitive outcomes of these studies was not considered the primary outcomes, while investigations involved heterogeneous populations with no standardization of tests or diagnostic criteria for defining cognitive decline or dementia, including its subtypes. In addition, these assessments were not adapted for target populations with socioeconomic and cultural disparities, while no supplementary methods such as imaging techniques were used to help corroborate outcomes.

The HYVET trial (*Hypertension in the very elderly Trial*) was a double-blind controlled trial using indapamide and perindopril in older elderly over 80 years of age which was cut short due to the benefits of using anti-hypertensives for reducing mortality and strokes. The HYVET-COG sub-study showed a non-significant reduction in dementia in the treated sub-group.^40^

**Recommendations** - The use of anti-hypertensives can reduce the risk of cognitive decline and dementia, including VD. Currently, there is insufficient evidence to recommend the use a specific class of anti-hypertensives (Level of evidence B).

### Statins

Dyslipidemic individuals have an elevated risk for developing dementia. Moreover, observational studies have shown that individuals treated with statins have lower risk for dementia. The preventive effect of statins in dementia hinges on their hypolipidemic effect. In addition, they exert anti-platelet, anti-thrombotic, anti-inflammatory effects and also have an impact on the formation of the beta amyloid protein, favoring the non-amyloidogenic path by alpha secretase.

Two randomized trials (HPS2002 and PROSPER2002) which included 26340 patients aged older than 70 years followed up for between 5 and 3.2 years, respectively, failed to confirm any effect on the incidence of dementia or cognitive decline. The HPS study using simvastatin, and the PROSPER with pravastatin, both found a significant reduction in LDL levels.^41^

The LEADe study was a randomized, double-blind, multi-center trial in 640 patients with mild to moderate AD assigned to receive atorvastatin 80 mg/day or placebo for 72 weeks. Results revealed no benefit in the treated group over placebo on cognition measured by the ADAS-Cog or on global functioning as measured by the ADCS-CGIC. No studies have assessed the role of statins in the management of VD.^42^

**Recommendations** - The use of statins in elderly individuals, subjects with vascular risk factors, is not recommended exclusively for the prevention or treatment of dementia (Level of evidence B).

### Diabetes

Recent studies have shown that diabetes is a risk factor for the manifestation of AD and VD. Besides raising the risk of vascular diseases, resistance to insulin has direct neuronal effects owing to glucose-induced toxicity (oxidative stress), abnormalities in homeostasis of cerebral insulin (amyloid metabolism) and microvascular abnormalities.

The occurrence of severe hypoglycemia in patients with type 2 DM can be associated to an increased risk of dementia, which rises with the number of hyperglycemic episodes.^43^

The ACCORD-MIND trial was designed to assess whether strict control of glycemia (target glycated hemoglobin lower than 6%) offers benefits over conventional levels (7 to 7.9%) for preventing cognitive decline.^44^ However, the ACCORD study showed that strict control of glycemia increased mortality and is contra-indicated in diabetic patients at high risk.^45^

Studies using PPAR-gamma (pioglitazone and rosiglitazone), independently of the presence of DM or glucose intolerance, have shown positive effects on cognition in AD. However, the study with rosiglitazone was discontinued as a result of increased mortality from cardiac problems in the treated group.

**Recommendations** - Strict control of glycemia (Glycated Hb < 6%) is not recommended for exclusively preventing cognitive decline in diabetic patients (Level of evidence B).

### Cardiac failure

Patients with low cardiac output have poor results on neuropsychological assessment, particularly regarding executive functions. In the Framingham trial, cardiac output was found to be associated to brain volume, where reduced cardiac function was related to faster brain aging, independently of vascular risk factors.^46^ Thus, if a reduction in systemic blood flow directly affects cerebral blood flow, then consequently, low cardiac output will reduce cerebral blood flow and contribute to encephalic compromise.^47,48^

The few studies available suggest that ACE inhibitors can have a beneficial effect on cerebral perfusion. In two such studies, patients with systolic dysfunction of the left ventricle (functional class NYHA III and IV) were treated with captopril^49,50^ leading to improved cerebral blood flow.

The use of ACE inhibitors has been associated to cognitive improvement, independently of basal blood pressure levels.^51^ A study assessing the impact of the use of anti-hypertensive drugs on cognition and cerebral vasoreactivity is currently underway.^52^

**Recommendations** - The treatment of potentially modifiable co-morbidities associated to cardiac failure in elderly patients (anemia, SAH, electrolyte abnormalities, hyperglycemia, hypoalbuminemia) can attenuate cognitive decline in this patient group (Level of evidence C). The use of ACE inhibitors can be recommended in patients with cardiac failure, independently of treatment to control blood pressure (Level of evidence C).

### Atrial fibrillation

Atrial fibrillation is a consistent risk for dementia in patients with history of cerebrovascular events, but this association is less clear in the broader AF patient population.^53^ Elderly patients with cognitive decline had shorter periods within the therapeutic window.^54^

**Recommendations** - In patients with AF and cognitive decline special attention should be paid to ensure adequate anticoagulation control (Level of evidence C).

### Sleep apnea

Studies have associated sleep apnea to cognitive decline in elderly without dementia (mildly affected) and also in more severe cases of apnea. Treatment of this sleep disturbance has a limited impact on cognition, and requires further exploration.

Sleep disturbance and day time sleepiness are risk factors for VD.^55^ Treatment of obstructive sleep apnea in AD patients has proven effective for improving some aspects of cognition and the quality of sleep in randomized studies.^56^

**Recommendations** - Obstructive sleep apnea must be investigated and treated in patients with dementia, and can yield some cognitive benefits (Level of evidence C).

### Tobacco use

Smokers are at greater risk of developing dementia in general, and more specifically, AD and VD. This finding has repeated in a number of observational studies.^57,58^ In the 23-year follow-up study by Rusanen et al. comparing 5367 individuals split into smokers of more than two packs a day and into non-smokers, showed the former group to be at higher risk for dementia (HR, 2.14;95% CI, 1.65-2.78), for AD (HR, 2.57;95% CI, 1.63-4.03), and for VD (HR, 2.72;95% CI, 1.20-6.18).^59^

The mechanisms involved include increased oxidative stress, greater inflammatory response, increase in the number of amyloid plaques and atherosclerosis. No controlled studies evaluating the impact of stopping smoking on cognition in later life have been published. Further studies are needed to determine at which point stopping smoking can lead to reduced risk.^36^

**Recommendations** - Stopping smoking should be recommended at any stage of life (Level of evidence C).

### Antiaggregants

In AD patients, the administration of acetylsalicylic acid increased the risk of hemorrhaging and led to no benefits in cognition. These patients should be given aspirin for vascular improvement.^60,61^

Regarding the prevention of dementias (AD and VD), epidemiological indices show that the use of non-hormonal anti-inflammatory drugs and acetylsalicylic acid reduces the risk of dementia.^62,63^

A randomized trial of aspirin and placebo spanning five years in individuals older than 50 years found no significant difference in cognition.^64^ The PROFESS study assessed acetylsalicylic acid plus dipyridamole versus clopidogrel and telmisartan, showing no difference between the impact of the two antiaggregants on cognition assessed by the Mini-Mental State Exam.^65^

**Recommendations** - The use of antiaggregants for primary prevention of cognitive decline and dementia is not recommended (Level of evidence B). The administration of acetylsalicylic acid is not indicated in AD patients for treating dementia, except when indicated for cardiovascular reasons (Level of evidence B).

### Carotid revascularization

Carotid revascularization in patients with symptomatic carotid stenosis has been proven to reduce risk of stroke recurrence and is considered an effective approach to secondary prevention in patients with large artery occlusive disease. However, the impact of carotid revascularization on cognitive performance remains controversial.

Numerous studies have assessed cognitive function after carotid revascularization either by surgical approach, carotid endarterectomy (CEA) or endovascular by carotid artery stenting (CAS). The outcomes reported by these studies have been conflicting. Many studies have shown no changes in cognition after the procedure, some have noted improvements while others have found decline.^66-70^

The likely reasons behind this disparity in results include differences in method and in the variables associated to patients. Notable methodological difference were types of tests employed, timing of test performance, and presence or otherwise of a control group. Patient-related variables included age and frequency of comorbidities (diabetes, previous stroke).

The majority of studies involved only a small number of patients. In addition, none of the studies estimated the power of the study *a priori*. These limitations may have led to results with low power for detecting differences in the outcomes assessed.

Only half of the studies assessing cognitive performance pre and post carotid revascularization were controlled. Of those studies incorporating a control group, the profile of the group was non-uniform with the inclusion of healthy individuals as well as cases of peripheral vascular surgery, orthopedic surgery, laminectomy, post angiography, among other conditions.

All the limitations outlined above mean the results of these studies must be interpreted with caution. Earlier reviews including studies published up to the year 2000, have suggested improvement in cognitive outcome after CEA,^70,71^ whereas a recent systematic review of trials with CEA and CAS, all published after 1990, showed that neither of the two procedures affected cognition.^72^

This latter review included 32 studies and analyzed results of tests assessing three cognitive domains: memory, executive function and language. However, no studies explored global assessment and dementia scores. Due to the heterogeneity of the methods used in different studies, the data from this review could not be pooled into a meta-analysis. According to the results of the review, carotid revascularization has no effect on cognitive performance in patients that did not have a stroke after the procedure.^72^

Three studies compared cognitive performance in patients treated with CEA versus angioplasty/CAS in sub-studies of two large clinical trials (CAVATAS and SPACE). None of these studies found any difference in cognition between the two groups (CEA *versus* Angioplasty/CAS).^73,74^

Data on the two procedures indicated for carotid revascularization will now be described, namely, CEA and CAS.

#### COGNITION AFTER CAROTID ENDARTERECTOMY (CEA)

Studies comparing cognitive performance before and after CEA have reported conflicting results. While the majority found no difference in pre and post procedure assessments, some studies showed deterioration while others reported improvement. Moreover, there was a lack of consistency in results on the different tests.^72^

Cognitive assessment after CEA was performed very early which may have led to underestimation of potential differences. Some studies have suggested that the potential effect of treatment on cognition tended to be more evident in assessments conducted later. Only one study had a follow-up period exceeding 1 year. This study showed cognitive decline over 3 years, evidenced by poorer scores on the mini mental state exam and worsening of motor abilities.^72^

#### COGNITION AFTER CAROTID ARTERY STENTING (CAS)

Studies assessing neuropsychological performance pre and post CAS also reported conflicting results. Half of the studies observed improvements in verbal memory. However, no difference in the language domain was detected, a domain which is harder to investigate in these patients. A potential effect of laterality has been proposed in several studies.^66,69^

The vast majority of the studies had extremely short follow-up periods. No data on patients' cognitive performance is available 6 months after undergoing the CAS.

The use of a protective device against embolisms can produce benefits in terms of cognition. Studies with CAS and no protective device (earlier studies) have found no changes in cognitive performance pre and post procedure. However, recent studies that more frequently used brain protection devices against embolism have shown cognitive improvement.^68,75^

A higher frequency of microembolism was noted in patients treated with CAS compared to those submitted to CEA. Nevertheless, no correlation between microembolism (detected by transcranial Doppler) and poorer cognitive outcome was evident.

**Recommendations** - Carotid revascularization using CEA or CAS in patients with symptomatic carotid stenosis has no effect on cognitive performance. Carotid revascularization should not be recommended for the purpose of preserving or improving cognitive function (Level C).

### Symptomatic pharmacological therapy

The measures listed below are exclusively intended for the management of VD cases, with approaches for AD treatment published in a separate article of the journal.

Currently, there is no Class I evidence to recommend specific symptomatic treatment for VD. A reduction in acetylcholine and choline acetyltransferase occurs in this dementia type, particularly in the presence of deep lesions, consequently impairing cholinergic pathways. Clinical trials using drugs with diverse mechanisms of action such as vasodilator effect, free-radical reducers, promoters of increased cerebral metabolism (through elevated glucose and oxygen extraction), with hemorheological properties, although based on justifiable theoretical presumptions given the physiopathological mechanism underlying VD, have failed to show efficacy in clinical practice. Potential beneficial effects can be attributed to inhibitors of cholinesterase.

No consistent benefits have been found for the treatment of VD in randomized trials based on the following drugs:

#### GINKGO BILOBA

All studies on this medication have involved dementia of various causes with no investigations dedicated to VD alone. Studies including a VD subgroup have adopted heterogeneous diagnostic criteria and produced inconsistent results.^76^

#### NICERGOLINE

No studies using this drugs focusing specifically on VD have been conducted. In those studies including a subgroup for this form of dementia, the criteria used for its diagnosis were poorly defined. All studies eligible for the Cochrane review consulted for this subject were early, having been published up to 1999. The results of these studies are inconclusive.^77^

#### VINPOCETINE

The few studies with a suitable methodology using this medication are early (published up to 1991) and include various forms of dementia, with heterogeneous diagnostic criteria. There is no evidence to justify the clinical use of this drug for the treatment of VD.^78^

#### CO-DERGOCRINE MESYLATE (HYDERGINE) AND OTHER ERGOLOID MESYLATES

only two methodologically sound trials on the use of co-dergocrine in VD are available. The results are inconclusive given the impossibility of comparing outcomes obtained by the two studies and owing to the small number of patients assessed.^79^

#### PIRACETAM

No clinical trials specifically designed for VD are available and the diagnostic criteria for the various forms of dementia in the studies assessed were highly heterogeneous. Therefore, based on current evidence, it can be concluded that this medication should not be used for the treatment of VD.^80^

**Recommendations** - The medications above should not be used in the treatment of VD (Level of evidence B).

#### PENTOXIFYLLINE

This agent is derived from xanthine which improves blood flow by increasing the deformability of erythrocytes through phosphodiesterase inhibition. A systematic review published in 2003 evidenced only four methodologically eligible clinical trials for assessment which used pentoxifylline for VD. The authors concluded that despite holding potential for inclusion in future studies (two studies showed performance improvement on some domains of the cognitive assessment in the pentoxifylline-treated group), the quality of the trials published to date cannot support its clinical use.^81^

**Recommendations** - Pentoxifylline is not recommended for the treatment of VD (Level of evidence U).

#### CITICOLINE

The mechanism of action of the drug Citicoline (Cytidinephosphocholine - CDP-choline) is unclear but it is believed to exert a repairing action through resynthesis of phospholipids after lesion and may act in cholinergic deficit.

A meta-analysis by the Cochrane group including seven articles showed some effect on memory, behavior and clinical global impression. However, the studies were heterogeneous in terms of doses used (ranging from 100 to 1000 mg/day), form of administration, inclusion criteria of subjects and outcome measures. Data published to date points to the need for randomized studies with a larger number of patients and longer follow-up periods.^82^

**Recommendations** - Currently available data is insufficient to be able to recommend citicoline for the prevention or management of patients with VD (Level C).

#### CEREBROLYSIN

This consists of a peptidergic compound with neurotrophic activity administered via the endovenous route. To date, a total of three randomized, double-blind clinical trials have been published using cerebrolysin. The study with the largest number of participants (242 individuals),^83^ showed a significant difference compared to placebo on scales measuring cognition (ADAS-Cog) and clinical impression (CIBIC+) (Class II). Studies involving larger cohorts are needed to confirm these observations.

**Recommendations** - Cerebrolysin is not recommended for the treatment of VD (Level of evidence C).

### Cholinesterase inhibitors and glutamate receptor antagonists

A meta-analysis study assessing cholinesterase inhibitors and memantine found a positive effect on the ADAS-Cog for all these medications ranging from a decrease of 1.10 for rivastigmine to -2.17 for donepezil. Only donepezil had an influence on the global clinical assessment measure. None of the drugs produced any behavioral or functional adverse effects except for donepezil at a dose of 10 mg/d. Benefits on cognition were small and of questionable clinical significance.^84^ Further studies are needed and their accuracy shall depend on the etiological mechanism implicated in VCI and the separation among its subtypes.

#### DONEPEZIL

This proved well tolerated and was shown to improve cognitive symptoms and functional abilities in VCI patients. Further, more extensive studies are required that assess the safety and efficacy of the drug for promoting the delay of cognitive decline.^85-89^

#### RIVASTIGMINE

Rivastigmine exerts specific activity in brain regions associated with executive dysfunction and reduced attention. Thus, there are theoretical grounds for believing that rivastigmine can be beneficial for treating VCI but no double-blind, placebo-controlled clinical trials are available.

#### GALANTAMINE

This drug is known to have limited efficacy in treating dementia secondary to vascular lesion. In mixed dementia (vascular lesions and AD), some evidence points to benefits for cognition.^95-100^

**Recommendations** - Current data is lacking to be able to justify the use of these substances in VD treatment. Assessments of VD sub-types are needed and use should be dedicated and targeted (Level of Evidence B). Benefits seem more marked in subcortical VD patient, according to the opinion of specialists involved in this consensus (Level of evidence C).

#### MEMANTINE

Memantine is a glutamate receptor antagonist (NMDA) which acts as a neuroprotective agent in dementia by blocking the neurotoxic activity of glutamate. The Cochrane review on the use of this medication in dementia incorporates only two clinical trials in patients solely with VD, while three trials included patients with AD and VD.^101^ Although results of the trials in VD patients showed slight improvement on ADAS-Cog scores, this did not reflect in improved cognitive performance from a clinical standpoint. Mobius et al. suggested that memantine can be more effective in patients with subcortical VD, explaining the low clinical repercussion in studies that encompassed various forms of VD.^101^ This hypothesis has yet to be confirmed by specific studies in well-defined groups with subcortical VD.

**Recommendations** - The data available is insufficient to justify the use of these substances in VD. Assessments of VD sub-types are needed and use should be dedicated and targeted (Level of Evidence B). Benefits seem more marked in subcortical VD patients, according to the opinion of specialists involved in the consensus (Level of evidence C).

### Calcium channel blockers

Two drugs of this class were tested for VD, namely, nimodipine and nicardipine.

A meta-analysis of 10 trials assessing the efficacy of nimodipine in randomized, double-blind, clinical trials of short duration (12 weeks) revealed global clinical improvement in nimodipine-treated patients. In randomized, double-blind, placebo-controlled trial, Pantoni et al. affirmed that patients with subcortical VD in use of nimodipine for 12 months had less marked decline on the MMSE and GDS compared with subjects given placebo.^103^ A persistent beneficial effect from use of nimodipine has yet to be satisfactorily demonstrated by long-term studies (longer than 12 weeks), weakening the arguments for its introduction in routine clinical practice.^104^

Two trials have been conducted on use of nicardipine in VD, both with methodological limitations which preclude the validation of the results. In the study conducted by the *Spanish Group of Nicardipine Study in Vascular Dementia*, favorable results were found for women only, as well as in patients concomitantly using platelet antiaggregants (Class II).^105^ The study by Gonzalez-Gonzalez and Lorano encompassing 5000 patients, showed improvement after six months of nicardipine use only among a sub-set of patients that presented with more severe condition at study baseline (Class III).^106^

**Recommendations** - Neither nimodipine nor nicardipine can be recommended for the treatment of VD (Level of evidence C).
